# Multi parametric biophysical assessment of treatment effects on xerotic skin

**DOI:** 10.1002/ski2.21

**Published:** 2021-03-21

**Authors:** H. Stettler, J. M. Crowther, M. Brandt, A. Boxshall, B. Lu, R. de Salvo, S. Laing, N. Hennighausen, S. Bielfeldt, P. Blenkiron

**Affiliations:** ^1^ Bayer Consumer Care AG Basel Switzerland; ^2^ JMC Scientific Consulting Ltd. Surrey UK; ^3^ proDERM GmbH Schenefeld/Hamburg Germany; ^4^ Illuminate Innovation Surrey UK; ^5^ Bayer Healthcare SAS Gaillard France

## Abstract

**Background:**

Topical moisturizing products are widely used to alleviate the problems associated with xerotic skin. Their use affects many properties of the stratum corneum (SC) in a complex and interrelated manner. The range of measurement techniques available to the researcher has increased in recent years. However, few studies have looked for correlations between the different techniques for assessing how aspects of xerotic skin change over time as a result of topical moisturizer usage.

**Objectives:**

A 3‐week in vivo study using an oil‐in‐water based moisturizing product and an untreated site was conducted to determine the clinical significance of and any correlations between a range of different approaches for the measurement of skin lipid content and also skin hydration and visual grading of dry skin.

**Methods:**

A range of traditional and more recently developed skin measurement techniques have been used to examine a variety of SC properties in normal and xerotic skin during topical moisturizer usage.

**Results:**

In vivo confocal Raman spectroscopy and analysis of SC lipids from tape strips both showed an increase in SC lipid level and organization after 3 weeks of moisturizer usage on xerotic skin. Hydration, measured both optically and electrically, also increased and skin barrier function improved, with strong correlations between the different measures of dryness being observed.

**Conclusions:**

Strong correlations were observed between the skin measurements for lipid assessment and skin hydration with regard to the assessment of xerotic skin, providing valuable new information for future in vivo clinical research into dry and atopic skin.

**Keywords**

biophysical assessment, skin barrier, skin hydration, topical moisturizers, Xerosis

## Graphical abstract

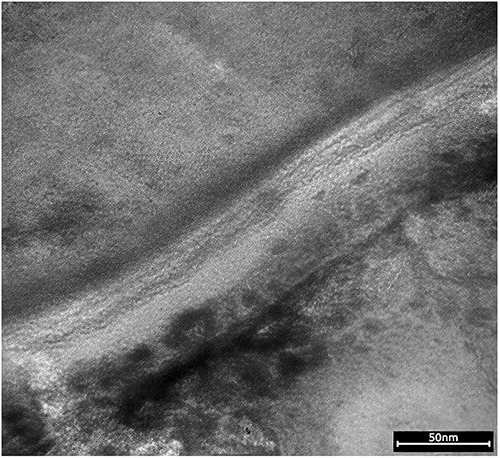

1

**What is already known about this topic?**

- The development of xerotic skin is a complex process and requires a multiparametric approach to describe.

**What does this study add?**

- New skin measurement techniques that are developed require comparison with existing methodologies to determine clinical relevance.

**What is the translational message**

- To assist with future clinical testing design, two approaches for stratum corneum lipid analysis (in vivo confocal Raman spectroscopy and Lipbarvis® lipid analysis) have been compared and contrasted. Also, a range of well‐established and novel skin hydration measures have been compared with each other and visual dry skin grading to determine what correlations exist.

## 2 INTRODUCTION

Dry, sensitive, xerotic skin affects up to around 50% of the world's population.^1^, ^2^ There are many factors that are involved in its development and a ‘dry skin cycle’ has been proposed for the evolution of cosmetic dry skin conditions.^3^ A typical feature of dry skin is defective corneocyte differentiation leading to the development of an immature stratum corneum (SC) with inferior barrier, hydration and desquamatory properties, and reduced levels of intercellular SC lipids.^4^, ^5^ It is the assessment of this wide range of parameters which is required when trying to fully understand the changes involved in returning to a healthy state from xerotic skin. Given the prevalence of xerotic skin, it is vital to provide accurate information to help with appropriate treatment.

Since the initial development of biophysical methods to measure properties of the SC,^6^, ^7^ their development has progressed at a rapid rate, now covering a wide range of skin properties. However the question as to the clinical significance of the readings that all of these devices give should always be considered.^8^, ^9^ Visual grading of dryness is still widely considered to be the gold standard for dryness investigation, however, it has become apparent that not all issues with skin are visible to the naked eye and that these ‘invisible dermatoses’ can only be detected by the use of instruments.^10^ The complexity of the relationship between what is observed as visibly dry skin, and what measurable properties of the skin have changed is discussed by Piérard.^11^ It is, therefore, important to test new biophysical methods against known standard techniques.

Recently, the authors reported the effects of using a topical oil‐in‐water based moisturizer on a range of skin parameters.^12^ The work presented here builds on the initial analysis reported in Stettler et al.^12^ to provide validation for a range of more recently developed skin measurement techniques against well‐established clinical skin assessment methods. The following study design was used; after a controlled washout period to stabilize the skin condition, subjects treated one leg with a topical moisturizer twice daily, while the other leg was left untreated. Measurements were taken at the beginning and end of the 3‐week treatment period from both the treated and untreated legs.

## 3 METHODS

### 3.1 Human study design

All subjects for the in vivo testing were recruited by proDERM GmbH, Hamburg, Germany. The study complied with the World Medical Association's Declaration of Helsinki (2000) concerning biomedical research involving human subjects, and the protocol approved by the Institutional Independent Review Board of proDERM. Sixteen subjects (average age 46.3 ± 4.3 years) were enrolled, consisting of Caucasian and Asian skin types. Inclusion criteria are given in the Supporting Information S1. All subjects underwent a 1 week washout phase, abstaining from moisturizer usage and using a standard wash product until the end of the study. One subject withdrew from the study and 15 subjects completed the study. Four adverse events were reported during the study (each one on a different subject) and all were mild in severity. They were not related to the use of the test product and were followed until resolution. Subjects received written informed consent and the test product ingredient list (shown in Supporting Information S2).

One cosmetic moisturizer (T), and one nontreatment control site (U) were assessed. The test cosmetic moisturizer (T) was an oil‐in‐water emulsion and the product ingredient list has been provided in Supporting Information S2 for the article. Subjects applied the products themselves twice daily to their lower leg (morning and evening) after being instructed in the correct application procedure. Treatment side for each subject was randomized and balanced between the left and right legs. Product dosage was based on a dose per unit area of approximately 2 mg/cm^2^. Subjects were told not to apply products to the skin on the morning of any days before any measurements were performed to not use any other moisturizers or wash products during the study, and to refrain from caffeinated drinks for at least 2 h before any measurement to exclude possible changes in skin water levels due to the pharmacological effect of caffeine.^13^

### 3.2 Skin assessment methods

Subjects were acclimatized for at least 30 min in a temperature‐controlled room (21 ± 1°C, humidity 50 ± 5%) prior to measurements.

In vivo confocal Raman spectroscopy (CRS) measurements were carried out using a gen2‐SCA Skin Analyzer (River Diagnostics). In the high wavenumber region between 2600 and 3800 cm^−1^ the concentration profiles were calculated to approximately 48 µm into the skin in 2‐µm steps. The following parameters were assessed: (i) thickness of SC/stratum disjunctum together (SC/SD) thickness was calculated as described in the work by Bielfeldt et al.^14^, (ii) lipid/protein ratio (between depths of 4–10 µm in the SC) as discussed in the work by Janssens et al.^15^

Skin capacitance measurements were taken using a CM825 Corneometer® (Courage & Khazaka). Five readings were taken in close proximity were taken for each site per time point. The highest and lowest were disregarded, and the remaining three readings averaged.

Transepidermal water loss (TEWL) was measured using a Tewameter® TM 300 (Courage & Khazaka). The probe was held in place for each measurement for 30 s. The values of the last 10 s (= 10 values) were averaged as the actual measurement value.

Dry skin images were collected using a Visioscan® VC 20plus (Courage & Khazaka). Exposure time for the images was 50 ms.

Two‐dimensional (2D) capacitance imaging of the skin was carried out using an Epsilon™ model E100 (Biox Systems Ltd.). The measurement settings were: capture mode, event trigger: 0.5, delay time: 2 s; average permittivity (ε) was measured. A circular mask 200 pixels in diameter was used to define the analysis area in a region where the device had good contact with the skin.

Mapping and visualization of the epidermal lipids in the SC was carried out using transmission electron microscopy (TEM) and the Lipbarvis® method (Microscopy Services Dähnhardt GmbH). Analysis was carried out to determine morphological changes (length of lipid lamella), and lipid content (ceramides 1 [EOS], 3 [NP] and 6 [NH], cholesterol and free fatty acids) using high‐performance thin layer chromatography as discussed in References^16^, ^17^, ^18^. On two neighbouring spots: one sample for morphological changes and one sample for lipid content were taken on each spot: two single carriers on the exact same area. Only the second carrier (approximate cell layers 5–8) was analyzed.

Visual grading of skin dryness was assessed using an Overall Dryness Skin Score and a trained skin assessor, as discussed in the work by Serup,^19^ based on the following criteria:

0 = Absent.
1 = Faint scaling, faint roughness and dull appearance.
2 = Small scales in combination with a few larger scales, slight roughness and whitish appearance.
3 = Small and larger scales uniformly distributed, definite roughness, possibly slight redness and possibly a few superficial cracks.
4 = Dominated by large scales, advanced roughness, redness present, eczematous changes and cracks.

### 3.3 Statistical analysis

A significance level of 0.05 (alpha) was chosen for statistical analysis. Comparisons of treatment and untreated sites were performed on differences to Baseline separately for each postapplication assessment time with paired *t*‐test. Comparisons of assessment times to Baseline were performed on raw data separately for each treatment with paired *t*‐test. This was carried out with SAS 9.4 for Windows (SAS Institute Inc.).

The comparison of skin hydration measurements with visual dry skin grading scores was carried out using a two‐way analysis of variance procedure, using treatment, site and subject as the main effects and baseline as covariate. Homogeneous groups were calculated using a significance level of 95% (*p* < 0.05). Errors were plotted as the least significant difference at the 95% confidence level. Correlation of the Corneometer® and Epsilon® hydration scores was carried out using a line of best fit. These assessments were performed in Statgraphics 18 for Windows (Statgraphics Technologies Inc.).

## 4 RESULTS

### 4.1 Summary of skin parameter differences in treated and xerotic skin states

A summary of the overall changes in skin properties for both the untreated (xerotic skin) and moisturizer treated skin sites at the end of Week 3 of the study are given in Table 1 (data provided from the work of Stettler et al.^12^). Significant changes were observed across the range of skin measures between the hydrated and xerotic skin sites. As would be expected, the treated skin was more hydrated than xerotic skin, had improved barrier function (TEWL) and demonstrated increased SC thickness. Treated skin also had increased levels of SC lipids and increased length of SC lipid lamellae.

**TABLE 1:** Differences from baseline for the treated and untreated (xerotic) sites for the measurements and assessments after 3 weeks of moisturizer usage (Columns A and B)

Measurement/assessment	A) Treated site difference to baseline	B) Untreated site difference to baseline	C) Difference between treated and untreated
Corneometer® CM825, a.u.	**10.58 ± 4.24, *p* < 0.001**	−1.71 ± 5.53, *p* = 0.250	**12.29 ± 6.70, *p* < 0.001**
Epsilon™ E100, permittivity ε	**3.69 ± 2.54, *p* < 0.001**	−0.29 ± 1.30, *p* = 0.404	**3.98 ± 2.31, *p* < 0.001**
Visioscan® VC20plus, scaliness score	−0.77 ± 2.217, *p* = 0.234	−0.92 ± 1.76, *p* = 0.084	−0.18 ± 2.17, *p* = 0.788
Visual dry skin grading, scale 0‐4	**−1.5, *p* < 0.001**	0.0, *p* = 1.000	**−1.5, *p* < 0.001**
TEWL Tewameter® TM300, g[H_2_O]m^−2^h^−1^	**−1.9 ± 1.90, *p* = 0.002**	−1.1 ± 2.20, *p* = 0.095	−0.9 ± 2.80, *p* = 0.258
CRS, SC/SD thickness, µm	**3.05 ± 3.84, *p* = 0.008**	0.29 ± 2.91, *p* = 0.702	**2.75 ± 4.53, *p* = 0.034**
CRS, lipid/Protein ratio, 4–10 µm average	**0.18 ± 0.16, *p* < 0.001**	−0.05 ± 0.18, *p* = 0.310	**0.23 ± 0.27, *p* = 0.006**
Lipbarvis®, lipid lamella length (LLL), nm/1000 nm^2^	**147.75 ± 28.27, *p* < 0.001**	8.01 ± 15.41, *p* = 0.064	**139.74 ± 29.39, *p* < 0.001**
Lipbarvis®, cholesterol, ng/133 mm^2^	**2.77 ± 2.10, *p* < 0.001**	−0.31 ± 1.23, *p* = 0.350	**3.07 ± 1.47, *p* < 0.001**
Lipbarvis®, free fatty acids, ng/133 mm^2^	**2.00 ± 1.74, *p* < 0.001**	−0.01 ± 1.33, *p* = 0.970	**2.01 ± 1.16, *p* < 0.001**
Lipbarvis®, ceramide EOS, ng/133 mm^2^	−0.03 ± 1.37, *p* = 0.941	−0.50 ± 0.92, *p* = 0.055	0.47 ± 1.10, *p* = 0.118
Lipbarvis®, ceramide NP, ng/133 mm^2^	0.07 ± 1.00, *p* = 0.780	−0.17 ± 0.63, *p* = 0.305	0.25 ± 0.68, *p* = 0.179
Lipbarvis®, ceramide NH, ng/133 mm^2^	**3.47 ± 2.73, *p* < 0.001**	**1.05 ± 1.62, *p* = 0.025**	**2.42 ± 1.56, *p* < 0.001**

*Note*: Shown are the differences between the treated and untreated sites after 3 weeks of moisturizer usage (Column C). Data has been given to two decimal points where available. Statistical differences (paired *t*‐test) with significance <0.05 are shown in bold. Data except for the Visioscan® VC20plus originally shared in the work by Stettler et al.^12^

Abbreviation: CRS, confocal Raman spectroscopy.

### 4.2 Skin lipid analysis

**FIGURE 1:**
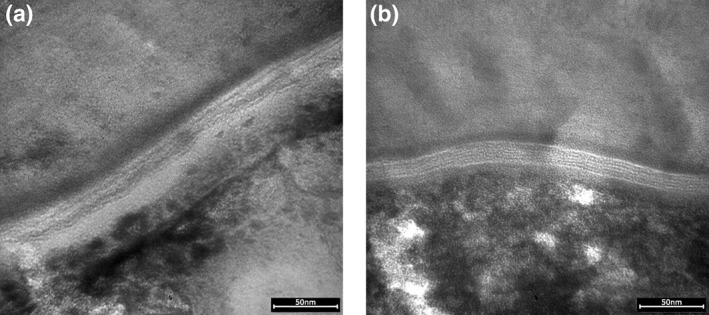
Transmission electron microscopy (TEM) images the lipid lamella structure of (a) xerotic, and (b) treated skin, collected during the study for Lipbarvis® assessment

Two different SC lipid assessments (CRS lipid/protein ratio and direct quantification from analysis of cyanoacrylate biopsies—Lipbarvis®) were carried out. The CRS lipid/protein ratio increased during the study for the treated site and was higher in treated skin compared to xerotic skin. Increased length of SC lipid lamellae and levels of cholesterol, free fatty acids and ceramide NH were observed after product usage. Typical TEM images obtained for xerotic and treated skin are given in Figure 1. Xerotic skin showed the expected disorganized intercellular SC lipids, while in treated, hydrated skin the SC lipid layers exhibited well defined lamellar organization.

Correlations between the changes in the lipid measures are given in Table 2. CRS measurement of the change in lipid/protein ratio was strongly correlated with the increase in length of lipid lamellae and cholesterol level derived from the Lipbarvis® analysis. Within the Lipbarvis® lipid parameters, the change in length of lipid lamellae strongly correlated with cholesterol, free fatty acid and ceramide NH level increases. Ceramide NP level change correlated with ceramide NH level increase. Ceramide EOS level change did not correlate with any of the other parameters.

**TABLE 2:** Correlation *p* values between the confocal Raman spectroscopy (CRS) measure of lipid/protein ratio and the Lipbarvis® lipid parameters

*p* Values for correlations	CRS lipid/protein ratio	Lipid lamella length	Cholesterol level	Free fatty acid level	Ceramide EOS level	Ceramide NP level	Ceramide NH level
CRS lipid/protein ratio		**0.0021**	**0.0043**	0.3106	0.5005	0.5502	0.1975
Lipid lamella length	**0.0021**		**0.0002**	**0.0041**	0.3468	0.2259	**0.0005**
Cholesterol level	**0.0043**	**0.0002**		**0.0086**	0.7058	0.1949	0.6021
Free fatty acid level	0.3106	**0.0041**	**0.0086**		0.3297	0.8259	0.7767
Ceramide EOS level	0.5005	0.3468	0.7058	0.3297		0.3740	0.3879
Ceramide NP level	0.5502	0.2259	0.1949	0.8259	0.3740		**0.0071**
Ceramide NH level	0.1975	**0.0005**	0.6021	0.7767	0.3879	**0.0071**	

**FIGURE 2:**
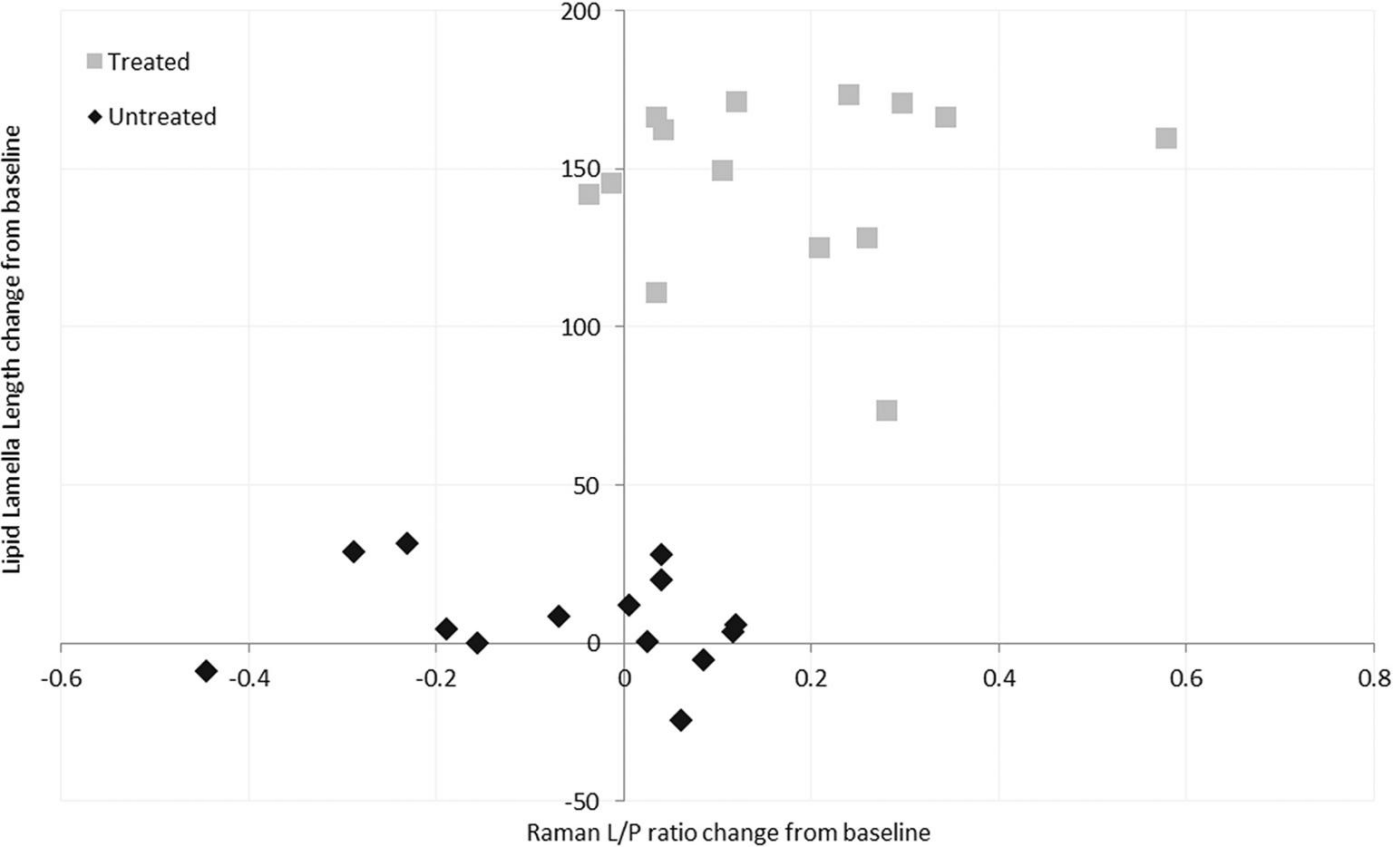
Comparison between the length of the SC lipid lamellae and CRS derived lipid to protein ratio for the untreated (xerotic) and treated skin sites. CRS, confocal Raman spectroscopy; SC, stratum corneum

The relationship between the change in CRS lipid/protein ratio and the change in the SC lipid lamella length for both the treated and untreated sites is shown in Figure 2 and shows two distinct clusters. At the treated site all the subjects showed increase SC lipid lamella length and all but two of the subjects had increased CRS‐derived lipid/protein ratio. For the xerotic untreated site, the subjects were clustered around the zero point for both the change in SC lipid lamella length and CRS lipid/protein ratio indicating little change from the baseline condition.

### 4.3 Visual dry skin grading and biophysical skin hydration measures

**FIGURE 3:**
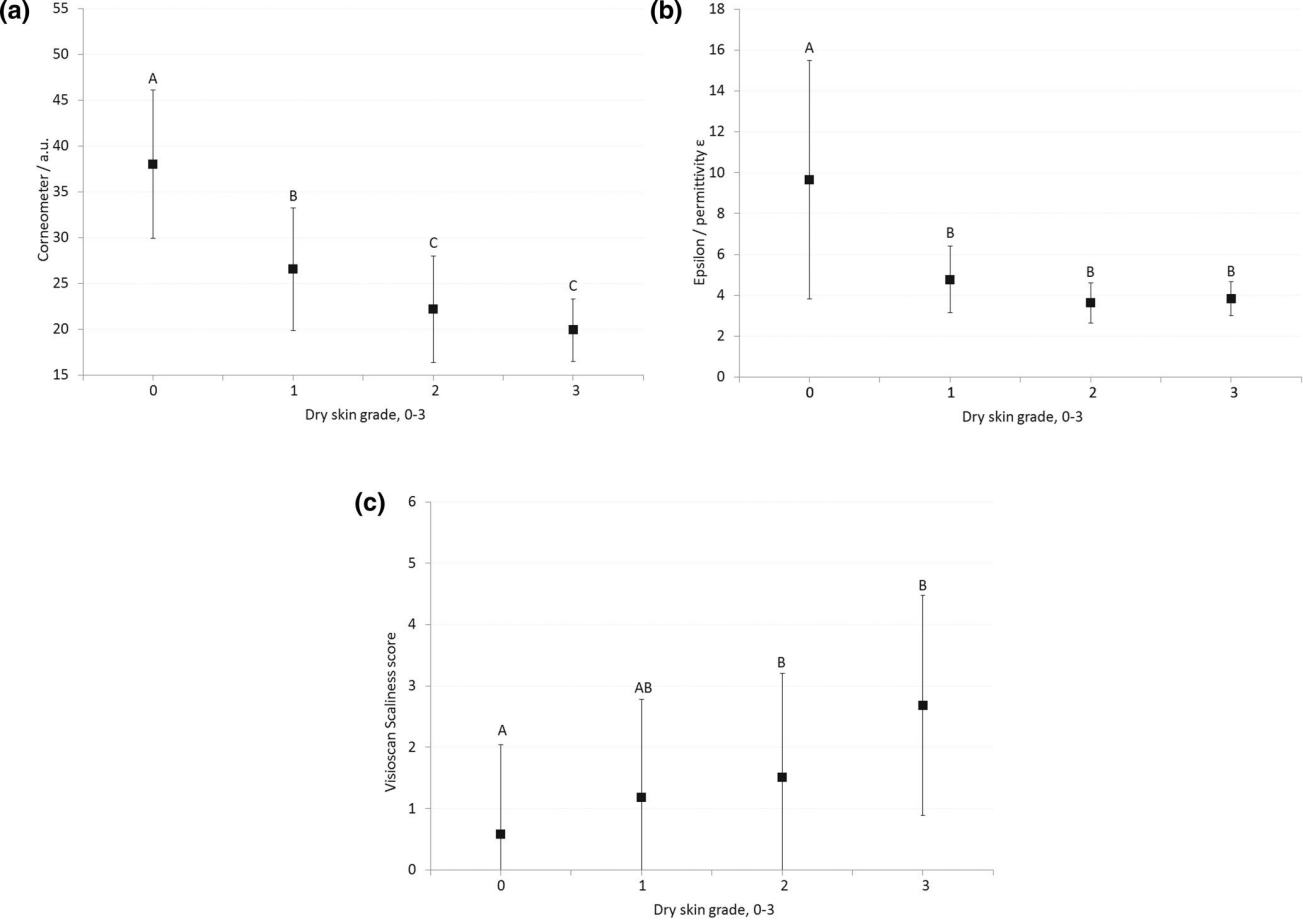
The relationship between visual grading of dryness and (a) Corneometer®, (b) Epsilon™ and (c) Visioscan® Scaliness scores. Each letter above the data points (‘A’, ‘B’ or ‘C’) indicate the homogeneous groups at 95% significance (*p* < 0.05) for the different measurement techniques

The comparisons between visual dryness grading scores and the three different biophysical measures of skin hydration are shown in Figure 3. Both the Corneometer® and Epsilon™ scores show a negative correlation with dry skin grading scores. The Corneometer® was able to differentiate between visual dryness scores of 0, 1 and 2, but not between grades of 2 and 3. The Epsilon™ was able to differentiate between dry skin grades of 0 and 1, but not between grades of 1, 2 and 3. The Visioscan® Scaliness score increased as the dry skin grading score increased, however did not discriminate between the different dry skin grades as strongly as the Corneometer® or Epsilon™ using the analysis approach carried out here.

**FIGURE 4:**
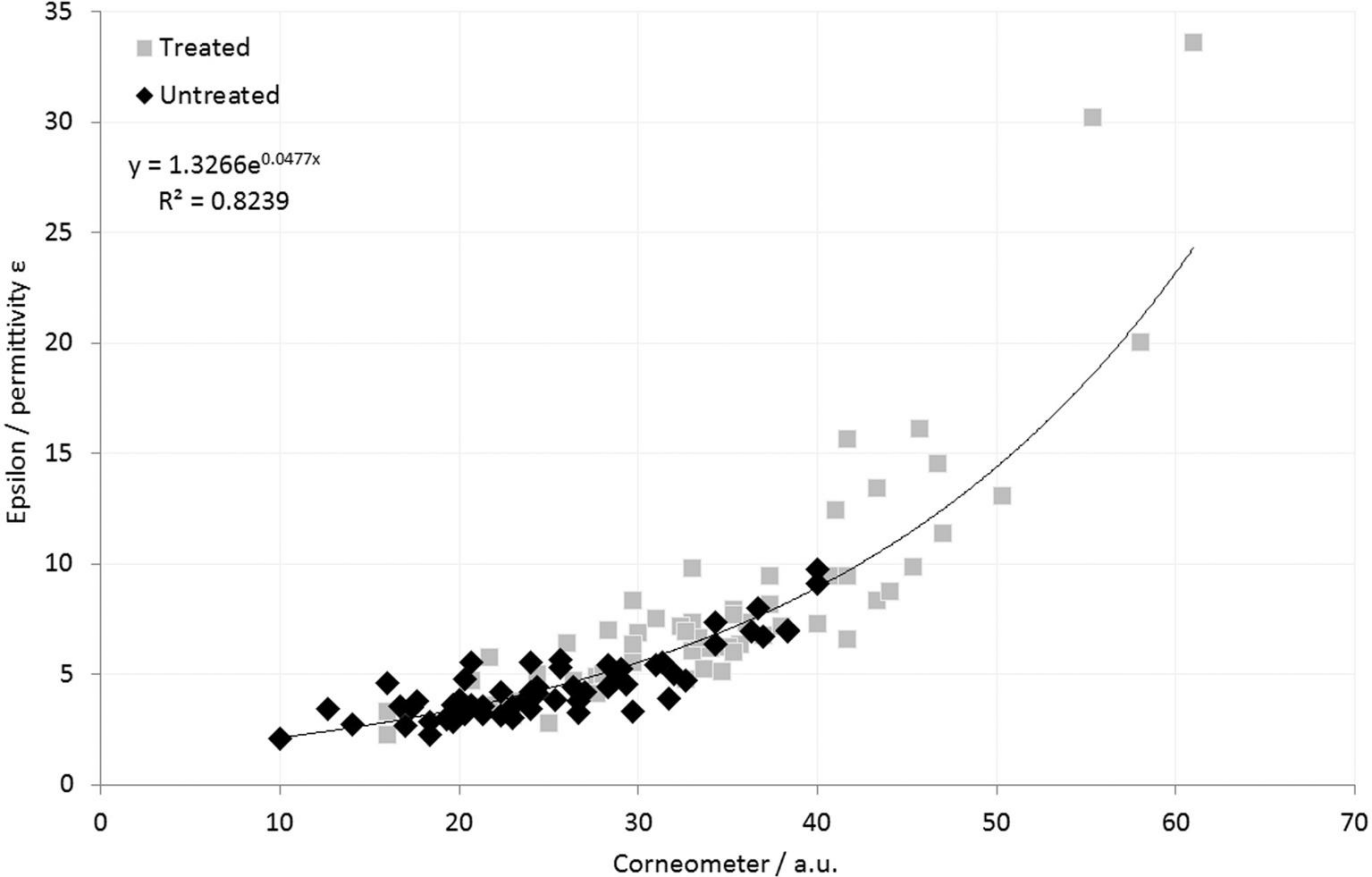
Correlation between Corneometer® and Epsilon™ scores, for both untreated (xerotic) and treated skin sites

The correlation between Corneometer® and Epsilon™ scores for both the treated and xerotic skin sites is shown in Figure 4. A line of best fit through the entire dataset for both the xerotic and treated skin sites is given, showing a strong correlation (*R* ^2^ = 0.8239).

## 5 DISCUSSION

Topical moisturizers are well known to alleviate dry skin when formulated appropriately. However, not all moisturizers have the same mode of action for how they affect the SC and the epidermis (for instance see Loden^20^). In the short term, moisturizers increase SC hydration,^20^, ^21^, ^22^, ^23^, ^24^, ^25^ and in the medium term they can improve desquamation.^26^, ^27^ With longer term usage, some products can improve the SC barrier^24^, ^25^, ^28^, ^29^, ^30^, ^31^ while others can compromise SC barrier function.^6^, ^7^, ^8^, ^9^, ^10^, ^31^, ^32^, ^33^, ^34^, ^35^, ^36^, ^37^ In vitro^38^, ^39^, ^40^, ^41^, ^42^ and in vivo^31^, ^43^, ^44^, ^45^ studies also demonstrated that moisturizing ingredients can influence SC thickness. Given this wide range of possible effects, it is important to look beyond simple hydration at a range of parameters when assessing the mechanisms by which topical moisturizers impact the skin.^11^, ^35^

After 3 weeks of products usage, skin hydration and barrier function improved. Levels of ceramide NH, cholesterol and free fatty acids increased, as did the ratio of lipid to protein in the SC and the average length of the intercellular SC lipid lamellae.

Ceramides, along with cholesterol and free fatty acids form the intercellular SC lipid matrix, and are major contributors to SC barrier function.^46^ Ceramides are the main component of the SC lipid bilayers comprising approximately 50% of SC lipid content by weight. Cholesterol and its derivatives and free fatty acids account for 25% and 10% to 20%, respectively.^47^ Reduced overall ceramide level and decreases in specific ceramides classes contributes to the incidence of xerosis.^48^ Reduction in ceramide NH level has been reported to correlate with increased incidence of skin roughness, dryness and scaliness on legs^49^ and is observed in subjects with psoriasis^46^ and atopic dermatitis,^50^, ^51^, ^52^, ^53^ highlighting its importance in SC formation and maintenance. In healthy skin, the SC intercellular lipids form a well ordered bilayer structure, comprising of hydrophobic and hydrophilic regions.^16^, ^38^, ^54^ In the study results presented here, while increases in the levels of ceramide EOS or NP were observed, they were not statistically significant, likely due to the small base size of the study.

While biophysical skin hydration assessment devices are widely used, assessment of the degree of dryness by a human grader is still widely undertaken as it takes into account both visual and tactile factors to determine an overall dryness rating.^19^ Visual dryness grading does, however, require a trained assessor and controlled lighting conditions in order to be successfully implemented. When it comes to interpreting measured values for skin hydration, as discussed by Piérard,^11^ it is easy to assume that all dry skin simply lacks water, when that is not always the case. Xerotic skin is lower in flexibility than hydrated skin, and this can reduce the ability of the skin to come in to close contact with the probe of a measurement device, resulting in a lower measured hydration score. In this work when compared to visual dry skin grades, the Corneometer® scores gave the best overall correlation, able to discriminate between the grades 0, 1 and 2, indicating its suitability for the assessment of skin dryness if a visual dry skin assessor is unavailable.

In addition to the single point type measures of skin hydration such as the Corneometer®, 2D arrays of sensors have been developed to allow for ‘hydration mapping’ of the skin. Based on the initial work in this area by Lévêque and Querleux with the development of the SkinChip device using fingerprint sensing technology^55^ commercial devices have been developed.^56^, ^57^ While the Epsilon™ 2D hydration measurement device used here was not as discriminatory to the visual dry skin grades as the Corneometer® in this work, it showed a similar trend. There was also a strong correlation between the Corneometer® and Epsilon™ readings. On the untreated sites the Corneometer® showed a greater dynamic range in the scores compared to the Epsilon™, the reason for this is not clear however could be due to the pressure with which the sensor comes into contact with the skin during use as discussed by Crowther.^58^ While some in vivo and in vitro correlations between electrical skin hydration measurement devices have been reported before,^58^, ^59^, ^60^ the authors believe this is the first time the correlation between the Corneometer® and Epsilon™ based on in vivo dry skin clinical assessment has been reported. In this study, the Epsilon™ was used in a basic analysis mode, with the values being generated based on the average measured permittivity across a region of the sensor and further data analysis is planned. In addition to the electrical measures of hydration in this study, optical assessment of dry skin was measured using the Visioscan® Scaliness parameter which works in the principle of fluorescence imaging. Because dry skin has not differentiated properly and fluoresces strongly under UVA illumination,^61^ skin scaliness can be seen as very bright pixels. Based on the proportion of white pixels in the image, the percentage of dry skin scaliness can be calculated. Visioscan® Scaliness scores have been previously reported to correlate with dry skin grades.^62^ A strong correlation between visual dry skin grading and the Visioscan® Scaliness score was not observed here. The reason for this is not clear, although the small base size of the study is likely a factor. It should also be noted that the work presented by Dobrev^62^ was performed with a different version of the device.

What can be said of the role of some of the ingredients in the test product formulation? Niacinamide has been reported to increase the synthesis of SC lipids in vitro including ceramides, cholesterol and free fatty acids along with improving TEWL after 4 weeks of using a formulation containing 2% niacinamide by Tanno et al.^63^ Niacinamide has also been reported to improve skin barrier function and increase SC thickness in a dose dependent manner,^31^ to improve corneocyte maturity,^64^ and to be active against acne.^65^ Dexpanthenol is a humectant and has been reported to improve SC hydration and to improve skin barrier function,^66^, ^67^, ^68^ play an important role in the synthesis of free fatty acids^69^ and in reducing keratinocyte growth factor overexpression which is important for correct differentiation.^69^, ^70^ In addition to its humectant properties, glycerin has also been shown to improve barrier function in damaged skin models using a TEWL measure.^25^, ^26^, ^71^ Isopropyl isostearate has been reported to improve orthorhombic lateral lipid packing in an in vitro skin model.^72^ The authors believe that a combination of ingredients in the formulation including niacinamide, dexpanthenol, isopropyl isostearate and glycerin are responsible for contributing to the overall improvements observed in the study discussed here.

Potential study limitations are discussed in Supporting Information S3.

In conclusion, significant differences in both the chemistry and structure of xerotic and topical moisturizer treated skin have been measured with a wide range of biophysical and skin assessment techniques. Two different approaches for the measurement of SC lipids (CRS and Lipbarvis®) have been compared and shown reduced levels of lipids in xerotic skin, in addition to shorter intercellular lipid lamellae indicating compromised barrier function. Three different approaches to skin hydration measurement have been compared with visual grading of dry skin, and been demonstrated to correlate with it to different degrees. Also, a 2D skin hydration mapping device (Epsilon™) has been shown to strongly correlate with a traditional and widely used skin hydration measurement device—the Corneometer® CM825—demonstrating the future potential for hydration mapping as a tool to provide information on skin hydration. We believe that this type of multiparametric assessment of xerotic skin can be used to provide a more complete description of the differences compared with moisturizer treated skin, as it enables a wide range of functional properties to be assessed simultaneously. The correlations that have been presented between the different measurement techniques will be of value to future in vivo clinical testing design with regards to the treatment of xerotic skin.

## CONFLICT OF INTERESTS

H. Stettler and R. de Salvo are employees of Bayer Consumer Care AG. P. Blenkiron and B. Lu are employees of Bayer Healthcare SAS. J. M. Crowther and A. Boxshall are consultants who have worked with Bayer Consumer Care AG and Bayer Healthcare SAS. M. Brandt, S. Laing, N. Hennighausen, and S. Bielfeldt are employees of proDERM GmbH where the study was carried out.

## Supporting information

Supplementary Material
