# Supplementary material for: Multi parametric biophysical assessment of treatment effects on xerotic skin
Source: Skin Health Dis. 2021 Mar 21;1(2):e21. doi: 10.1002/ski2.21 (PMC9060006; doi:10.1002/ski2.21)
Supplement: Supplementary file 1 — Supplementary Material [file SKI2-1-e21-s001.docx]

Multi parametric biophysical assessment of treatment effects on xerotic skin

H. Stettler^1^, J. M. Crowther^2^, M. Brandt^3^, A. Boxshall^4^, B. Lu^5^, R. de Salvo^1^, S. Laing^3^, N. Hennighausen^3^, S. Bielfeldt^3^, P. Blenkiron^5^

^1^Bayer Consumer Care AG, CH-4052 Basel, Switzerland;

^2^JMC Scientific Consulting Ltd, Egham, Surrey, TW20 8LL, UK;

^3^proDERM GmbH, 22869 Schenefeld/Hamburg, Germany;

^4^Illuminate Innovation, Egham, Surrey, TW20 9RN, UK;

^5^Bayer Healthcare SAS, 74240 Gaillard, France

Corresponding Author:

Dr Hans Stettler

Senior Medical Manager Dermatology

Bayer Consumer Care AG

Tel: +41 58 272 77 45

Fax: +41 58 272 79 02

E-mail: hans.stettler@bayer.com

**Supplementary information**

S1. Inclusion criteria for subject recruitment;

• Signed-off written Informed Consent to participate in the study.

• Willingness to actively participate in the study and to come to the scheduled visits.

• Healthy female.

• From 40 to 55 years of age.

• Pre-menopausal.

• Healthy skin in the test area.

• BMI < 30.

• Regular user of emollients to treat the dry skin condition.

• Fitzpatrick types I – III.

• Negative pregnancy test for women of childbearing potential.

• Uniform skin color and no erythema or dark pigmentation in the test area.

• Dry Legs (Overall Dry Skin Score 1-3) at Day 1.

• Corneometer® values on lower legs at Day 1 < 35 a.u.

S2. The ingredients contained within the test product (T) were:

Aqua, Butyrospermum Parkii Butter, Glycerin, Caprylic/Capric Triglyceride, Isopropyl Isostearate, Niacinamide, 1,2-Hexanediol, Cera Alba, Dexpanthenol, Squalane, Polyglyceryl-6 Distearate, Cetearyl Alcohol, Isosorbide Dicaprylate, Jojoba Esters, Tocopheryl Acetate, Glyceryl Stearate Citrate, Behenyl Alcohol, Polyglyceryl-3 Beeswax, Cetyl Alcohol, Argania Spinosa Kernel Oil, Xanthan Gum, Acrylates/C10-30 Alkyl Acrylate Crosspolymer, Citric Acid.

S3. Limitations of the study.

There were limitations with the study, which will now be discussed. The base size of the study was relatively small. This was done as a wide range of measurement methods and assessments were being employed. The study size was kept relatively small to ensure that the study could be adequately controlled. Though a larger base size would have been preferred the authors believe this an acceptable compromise, and strong statistical breaks were still observed for the majority of the measures. Although there was a significant improvement in SC barrier function (TEWL) for the treated site compared to the baseline, which was not observed for the untreated test site, the improvement in barrier function was not as well defined as would have been expected for the test product based on assessment of the formulation, and comparison with other products with similar levels of niacinamide [Crowther JM, Matts PJ, Kaczvinsky JR. Changes in Stratum Corneum thickness, water gradients and hydration by moisturizers. In: Treatment of dry skin syndrome (Loden M, Maibach HI, eds), Berlin, Germany: Springer-Verlag, 2012; 545-560]. There was a significant change in the weather during the study; at the start of the study the weather was relatively warm and humid (>80% humidity) for the time of year that the study was run, and during the study humidity dropped to <30% and the temperature also dropped significantly. The baseline scores for TEWL were higher than the rest of the study. The authors believe that this high baseline TEWL coupled with the gradual drop in TEWL for the untreated site as the ambient humidity dropped, contributed to the weaker than expected barrier improvement obtained after 3 weeks of product usage. After initial training in the correct amount of product to apply, and the site to which it should be applied, the subjects were responsible for dosing and applying the product themselves throughout the study. Subject compliance was checked by weighing the product tubes at the end of each week, and although there was some variability in the amount applied, on average the dose was equal to or greater than the target dose of 2 mg cm^-2^. As such the authors do not believe that self-dosing presented an issue for the study design or execution.
